# Non-Pharmacological Interventions for Post-Stroke Fatigue: Systematic Review and Network Meta-Analysis

**DOI:** 10.3390/jcm9030621

**Published:** 2020-02-25

**Authors:** Ya Su, Michiko Yuki, Mika Otsuki

**Affiliations:** 1 Graduate School of Health Sciences, Hokkaido University, Sapporo, Hokkaido 060-0812, Japan; swanvivi@eis.hokudai.ac.jp; 2 Faculty of Health Sciences, Hokkaido University, Sapporo, Hokkaido 060-0812, Japan; lasteroideb612@pop.med.hokudai.ac.jp

**Keywords:** stroke, fatigue, nonpharmacological interventions, randomized controlled trials, network meta-analysis

## Abstract

Post-stroke fatigue (PSF) is one of the most serious sequelae, which often interferes with the rehabilitation process and impairs the functional recovery of patients. Due to insufficient evidence, it is unclear which specific pharmacological interventions should be recommended. Therefore, in this paper, we compare the effectiveness of non-pharmacological interventions in PSF. A systematic review and network meta-analysis of randomized controlled trials were performed using EMBASE, MEDLINE, CINAHL, Cochrane library, ClinicalTrials.gov, CNKI, and CQVIP, from inception to January 2018, in the English and Chinese languages. RCTs involving different non-pharmacological interventions for PSF with an outcome of fatigue measured using the Fatigue Severity Scale were included. Multiple intervention comparisons based on a Bayesian network are used to compare the relative effects of all included interventions. Ten RCTs with eight PSF non-pharmacological interventions were identified, comprising 777 participants. For effectiveness, most interventions did not significantly differ from one another. The cumulative probabilities of the best non-pharmacological intervention for fatigue reduction included Community Health Management (CHM), followed by Traditional Chinese Medicine (TCM) and Cognitive Behavioral Therapy (CBT). Network meta-analysis based on data from the selected RCTs indicated that the eight PSF non-pharmacological interventions shared equivalent efficacy, but CHM, TCM, and CBT showed potentially better efficacy. In the future, fatigue needs to be recognized and more accurate assessment methods for PSF are required for diagnosis and to develop more effective clinical interventions.

## 1. Introduction

Fatigue is a common and long-standing complication after stroke. The prevalence of post-stroke fatigue (PSF) ranges from 25% to 85% [1]. The first report of fatigue after stroke, published in 1999, stated that 40% of stroke patients reported fatigue as one of their most serious sequelae [2]. PSF often limits the rehabilitation process and impairs the functional recovery of patients [3], and can also indirectly affect patients’ psychological outcomes and quality of life. PSF has also been closely related to prognosis and mortality [4]. As there is currently no specific measurement to identify fatigue and the signs of fatigue are not always obvious to outsiders, it may be difficult to understand how a patient is feeling. Thus, early detection and effective interventions are particularly important. Recently, PSF has gained increasing attention from researchers. The Canadian Stroke Best Practice Recommendations, the first best practice recommendations for PSF, were published in 2015 [5]; further, in 2016, the top 10 published research priorities specific to stroke nursing identified managing fatigue as a top research priority [6]. However, fatigue still does not receive enough attention in patients after stroke, making the management of fatigue in patients after stroke difficult and directly affecting their prognosis.

Fatigue is a symptom commonly experienced in the general population. “Nonpathological fatigue” describes a state of general tiredness if it lasts fewer than 3 months and has an identifiable cause, which is related to lifestyle or overexertion and can be ameliorated by rest. In contrast, “pathological fatigue” is experienced in many people with chronic illness, which has a longer duration, is difficult to treat, and can cause severe impairments to an individual’s functional activity and quality of life [7]. Nonpathological fatigue is mostly acute, but pathological fatigue is chronic in nature. PSF is not like typical tiredness, in that it does not always improve with rest. After a stroke, people may lack energy or strength, feel constantly weary or tired, and may not feel in control of their recovery. Marleen H. de Groot et al. defined PSF as a feeling of physical tiredness and lack of energy, described as pathologic, abnormal, excessive, chronic, persistent, or problematic [8]. Joanna Lynch et al. defined PSF for community and hospital patients. PSF in Community patients is defined as at least a 2-week period over the past month when the patient has experienced fatigue, lack of energy, or an increased need to rest every or nearly every day, leading to difficulty in taking part in everyday activities. In Hospital patients, PSF is defined when the patient has experienced fatigue, a lack of energy, or an increased need to rest every day or nearly every day since their stroke. Fatigue leads to difficulty in taking part in everyday activities (for inpatients, this may include therapy and may include the need to terminate an activity early due to fatigue) [9]. PSF is commonly measured using general fatigue scales, such as the Fatigue Severity Scale (FSS) and Checklist of Individual Strength (CIS), as shown in Table 1 [10,11,12,13,14,15,16,17,18,19,20,21]. The prevalence of PSF ranges from 25% to 85%, and is likely reflected by different patient populations as well as different measurement tools [1]. However, subjective general scales must be used, as there is currently no objective method to identify PSF.

Pharmacological intervention has been reported to improve PSF, such as Tirilazad Mesylate, Modafinil, and OSU6162. However, there is currently insufficient evidence to determine a specific pharmacological intervention for PSF, and pharmacological management is far from satisfactory. Moreover, there is a lack of systematic nursing management intervention for PSF [22]. Therefore, evidence-based medicine for PSF patients is required to provide a theoretical basis for prevention, and treatment with targeted health management programs are required to improve the quality of life of patients with fatigue after stroke. In this study, we aim to compare the effectiveness of non-pharmacological interventions for PSF to provide evidence for healthcare providers. Network meta-analyses (NMA), enabling the comparison of multiple interventions to incorporate clinical evidence from both direct and indirect treatment comparisons in a network of treatments and associated trials, is a valuable tool in comparative effectiveness research [23]. To the best of our knowledge, this is the first study using NMA for a multiple intervention comparison of the currently available methods to determine the effectiveness of non-pharmacological interventions in PSF. To provide effective support for stroke patients, it is necessary to first understand the effectiveness of non-pharmacological interventions.

## 2. Materials and Methods

This systematic review and network meta-analysis was conducted in accordance with the PRISMA statement extension for NMA [24]. We followed a pre-specified protocol registered at PROSPERO (CRD42018105983).

### 2.1. Inclusion and Exclusion Criteria

Only RCTs including outcome using fatigue score measured by FSS were used. We considered that differences in the prevalence of PSF are likely reflected by different measurement tools, in order to minimize the bias induced by the measurement of the outcome. Our inclusion criteria were any outcome of fatigue measurement using FSS, as FSS is a widely accepted and used scale to measure fatigue in stroke populations. We included any patients diagnosed with ischemic or hemorrhagic stroke, as diagnosed by MRI or CT, and no age or gender limitations were considered. The control group was defined by treatment as usual, including usual treatment, nursing, and rehabilitation, which we called “as usual” (AU). The intervention group was defined as additional provided non-pharmacological interventions based on usual treatment, where non-pharmacological intervention denotes the management of PSF without medications. The outcome was the patient’s degree of fatigue pre- and post-intervention using the FSS scale.

### 2.2. Data Search and Selection

We searched EMBASE, MEDLINE, CINAHL, Cochrane library, ClinicalTrials.gov, CNKI, and CQVIP from inception to Jan. 2018, using the English and Chinese languages, and updated the search to 2019. Our search terms are shown in Table A1. Two reviewers (Y.S. and M.O.) independently read the titles and abstracts identified by the search, then screened the full text manuscripts of potentially relevant references. Any eligibility disagreements were decided by discussing with a third reviewer (M.Y.).

### 2.3. Data Extraction and Quality Assessment

Data extraction details included identification of the study, methods of study design, participant characteristics, interventions, outcome measures, and results. Data from baseline and endpoint of fatigue score were included in the results. If results included multiple post-intervention and follow-up scores, we chose the last follow-up score as the endpoint score.

The risk of bias of the included RCTs was assessed based on the Cochrane tool using the Review Manager version 5.3 (The Nordic Cochrane Centre, The Cochrane Collaboration, Copenhagen, Denmark), with six assessment domains: Selection bias, performance bias, detection bias, attrition bias, reporting bias, and other bias. For each study, the classification of “low risk” was shown in green, “unclear risk” was shown in yellow, and “high risk” was shown in red.

### 2.4. Statistical Analysis

First, a network plot for every intervention was drawn used using the STATA version 14.0 (StataCorp LP. College Station, TX, USA). Second, we conducted pair-wise meta-analyses with a random effects model to synthesize studies comparing the intervention with control (AU). The results were reported as pooled mean difference (MD) with the corresponding 95% confidence interval (CI). Statistical heterogeneity across studies was assessed using a forest plot and the inconsistency statistic (I^2^). Statistical significance was regarded as *p* < 0.05. All calculations were performed using Review Manager version 5.3 (The Nordic Cochrane Centre, The Cochrane Collaboration, Copenhagen, Denmark). Third, mixed comparisons were carried out on direct and indirect evidence. We conducted Bayesian NMA using the Markov Chain Monte Carlo random effects model in Aggregate Data Drug Information System (ADDIS) version 1.16.8 (Drugis, Groningen, NL). We networked the translated FSS outcomes within studies and specified the relations among the MD across studies making different comparisons, as previously reported. This method combines direct and indirect evidence for any given pair of interventions. We used *p* < 0.05 and 95% CI beyond the null value to assess significance. We also calculated the inconsistency factor (IF) and 95% CI to evaluate the inconsistency of each closed loop, with the IF close to 0. In additional, the random effects variance and inconsistency variance were roughly equal, which is considered to be less inconsistent. Furthermore, we assessed the probability that each intervention was the most efficacious, the second best, the third best, and so on, by calculating the MD of each treatment group, compared with arbitrary common controls, and counting the proportion of iterations of the Markov chain of the MD ranking in treatments.

## 3. Results

**Figure 1:**
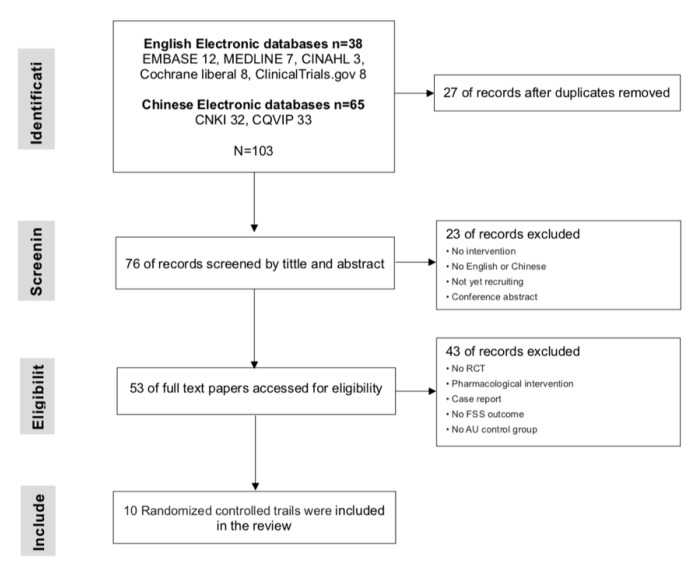
Flow diagram. FSS = Fatigue Severity Scale; RCT = randomized controlled trails; AU = as usual (treatment, nursing, rehabilitation, education).

**Figure 2:**
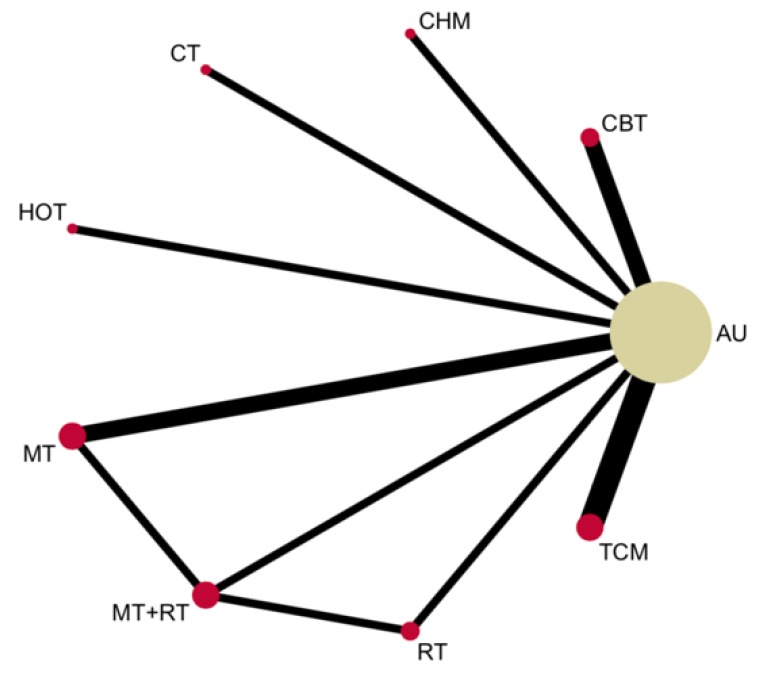
Network plot for each intervention. The size of the nodes is proportional to the sample size of each intervention and the thickness of the lines proportional to the number of trails available. AU = treatment, nursing, rehabilitation, education as usual; CBT = cognitive behavioral therapy; CHM = community health management; CT = circuit training; HOT = hyperbaric oxygen therapy; MT = music therapy; RT = respiratory training; TCM = traditional Chinese medicine.

Studies were selected by following PRISMA guidelines [23]. Figure 1 presents a flow diagram showing the searching and selection process for this systematic review. This systematic review identified 103 records and, ultimately, included 10 RCTs which compared non-pharmacological interventions in the PSF population. A total of 777 patients from the 10 selected RCTs were included. The population study sizes varied from 15 to 242, median age ranged from 47 to 69 years, and disease duration ranged from 2 weeks to 27 months. The studies were conducted in Australia, the Netherlands, and China, and the publication dates ranged from 2012 to 2018. We updated the search to 2019 and found 4 pharmacological intervention trials that were excluded. The characteristics of each trial are shown in Table A2. Eight non-pharmacological interventions were used for the analyses, and the network plot for each intervention is shown in Figure 2.

### 3.1. Type of Intervention

#### 3.1.1. Community Health Management (CHM)

One study assigned 90 patients to CHM and control (AU) groups at random [25]. The CHM team consisted of 10 nurses, one neurology chief physician, two rehabilitation physicians, and one psychological consultant. The CHM team assessed patients the day before discharge, provided a stroke management manual for patients, and followed up (by telephone) at 1, 2, 5, 8, and 12 weeks after discharge. In the present study, the health management of stroke patients included drug management, fatigue education, community activities, and psychological care. After implementing CHM, the FSS of the CHM group were lower than those of the control group (AU) and pre-intervention. This indicates that conducting community-based post-stroke health management can effectively prevent the occurrence of PSF, reduce the incidence of PSF, and improve the quality of life in stroke patients.

#### 3.1.2. Traditional Chinese Medicine (TCM)

Three studies showed that TCM intervention could improve fatigue after stroke [26,27,28]. The first study [26] used acupuncture at Baihui and Sishencong, using 200 rpm for 2 min per needle and leaving the needle for 30 min, once a day for five days a week for a total of four weeks. In the second study [27], moxibustion treatment was combined with intermediate frequency electric acupoint massage for 15 days. Moxibustion treatment combined with massage was performed once per day, which involved selecting acupoints (e.g., Baihui, Shenque, and Zusanli acupoints) for moxibustion, using 3–5 acupoints each time for 15–20 min per acupoint. The third study [28] investigated transcutaneous acupoint electrical nerve stimulation targeting Zusanli, Neiguan, Guanyuan, Pishu, and Qihai acupoints using Han’s acupoint stimulator for 30 min, once a day for a total of two weeks.

#### 3.1.3. Cognitive Behavioral Therapy (CBT)

Two studies investigated CBT [29,30]. The intervention by Nguyen et al. [29] used a standardized CBT treatment manual comprised of six modules addressing fatigue over eight individual therapy sessions. Treatment encompassed the psychoeducation CBT framework, reorganization of daily schedules, energy conservation, cognitive restructuring, sleep interventions, strategies for physical and mental fatigue, and review techniques for relapse prevention. The second study [30] included four CBT sessions based on problem solving methods, relaxation training, education, follow-up, and support by telephone. The study concluded that cognitive behavioral intervention based on problem solving could effectively improve fatigue after stroke, as well as medication compliance to help patients recover.

#### 3.1.4. Respiratory Therapy (RT) and Music Therapy (MT)

Two studies investigated RT and MT [31,32]. The MT-based study [31] included 40 patients with usual nursing and selected the appropriate music and volume, depending on the patient’s condition; patients underwent MT for 30 min, once per day for five days a week, for a total of eight weeks. After the intervention, fatigue scores were lower than the AU group and quality of life scores were higher. The other one study [32] included 80 patients divided into four groups: MT, RT, RT + MT, and AU. The RT group received rehabilitation using a breathing exercise for 15 min twice a day, for five days a week; the MT group received rehabilitation using music therapy for 30 min once per day for five days a week; the RT + MT group received both therapies five days a week. After eight weeks of intervention, the RT + MT group had the lowest FSS scores and the AU control group had the highest FSS scores.

#### 3.1.5. Circuit Training (CT)

One study involved 250 patients undergoing CT [33]. The intervention included a 90 min graded task-oriented CT program twice a week over 12-weeks (24 sessions). It included four stages: Warm up (15 min), CT (60 min), evaluation and a short break (10 min), and a group game (15 min). The study found that CT improved walking speed, stair walking, and walking distance, but showed no significant effects in fatigue after stroke; possibly because the patients had low average baseline fatigue and depression levels.

#### 3.1.6. Hyperbaric Oxygen Therapy (HOT)

One study of 62 patients undergoing HOT was found [34]. Patients absorbed pure oxygen once a day for 20 min through a mask, and the procedure was repeated three times with a rest time of 5 min in between. The study showed that, after a four-week intervention, the AU group showed aggravation of PSF; however, the HOT group showed no significant difference in FSS scores.

### 3.2. Assessment of Risk of Bias

**Figure 3:**
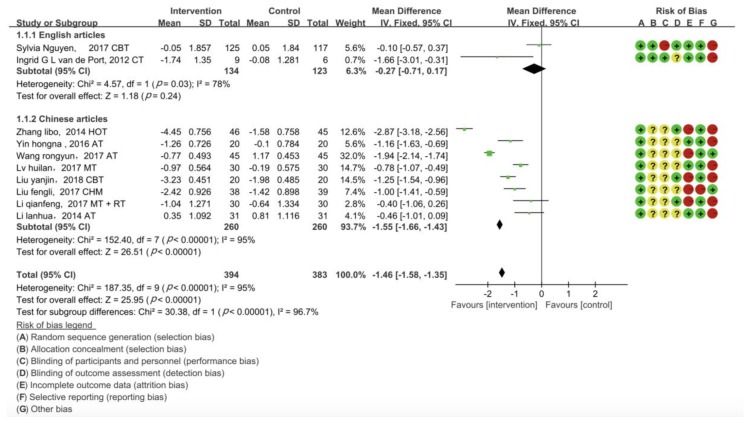
Forest plots and assessment of risk of bias. Horizontal lines correspond to study-specific MD and 95% CI. The area of the square reflects study-specific weight. The diamond represents pooled results of MD and 95% CI. (1.1.1) Articles in English and (1.1.2) Articles in Chinese. For each study, the selection bias, performance bias, detection bias, attrition bias, reporting bias, and other biases were assessed at “low risk” if shown in green, “unclear risk” if shown in yellow, and “high risk” if shown in red.

We summarize the results of our assessment of the risk of bias for the included studies in Figure 3. All study designs were RCTs, and a high risk of bias was not found in the design of any studies. However, concealment of allocation was difficult to assess in eight studies, due to poor reporting. There were 10 (100%) RCTs with a low risk of bias in random sequence generation and 9 (90%) with a low risk of bias in selective reporting. One RCT showed low risk and high risk of bias in participants and outcome assessment, respectively. Blinding of outcome assessment was difficult to assess in six studies, due to poor reporting. As for incomplete outcomes, five studies had a low risk of bias.

### 3.3. Pair-Wise Meta-Analysis

Figure 3 summarizes the outcomes, showing that CBT, CHM, HOT, MT, RT, and MT + RT interventions were significantly better than the control treatment (AU). TCM with transcutaneous acupoint electrical nerve stimulation and moxibustion combined with intermediate frequency electric acupoint massage were also significantly better than the control treatment. However, TCM with acupuncture at Baihui and Sishencong acupoints was not significantly different from AU (−0.40 (−1.07, 0.27)). Direct meta-analysis of the English articles’ subgroup showed significant heterogeneity between trials (I^2^ = 78%, degrees of freedom (df) = 1, *p* = 0.03). The Chinese articles subgroup showed significant heterogeneity between trials (I^2^ = 95%, df = 7, *p* < 0.00001).

### 3.4. Network Meta-Analyses for Interventions

We established a network for non-pharmacological interventions in PSF. Table 2 summarizes the results of the network meta-analysis regarding the reduction of fatigue after stroke by FSS. The results show that TCM, CT, CBT, CHM, HOT, MT, RT, MT + RT, and eight PSF non-pharmacological interventions were not statistically different in MD for FSS reduction scores.

**Figure A1:**
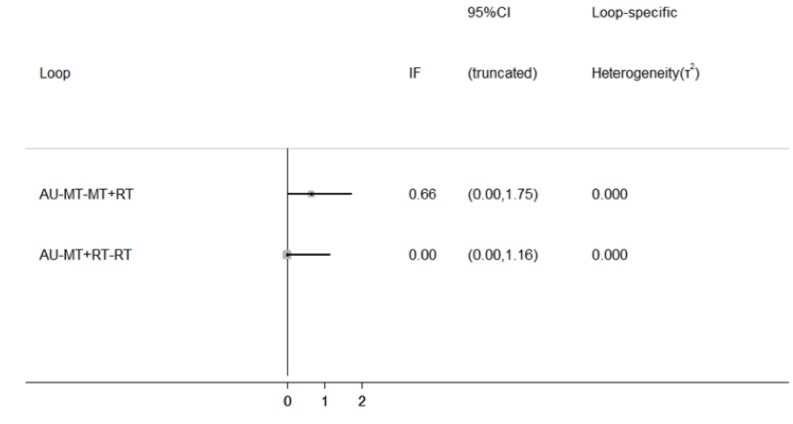
Inconsistency test.

We checked for inconsistency, where the IF was 0.00 and 0.66 and, thus, was close to 0 (Figure A1). In addition, the random effects variance (1.28 (0.63, 2.55)) and the inconsistency variance (1.27 (0.64, 2.55)) were roughly equal, which is considered to be less inconsistent.

### 3.5. Rank Probability of Interventions

**Figure 4:**
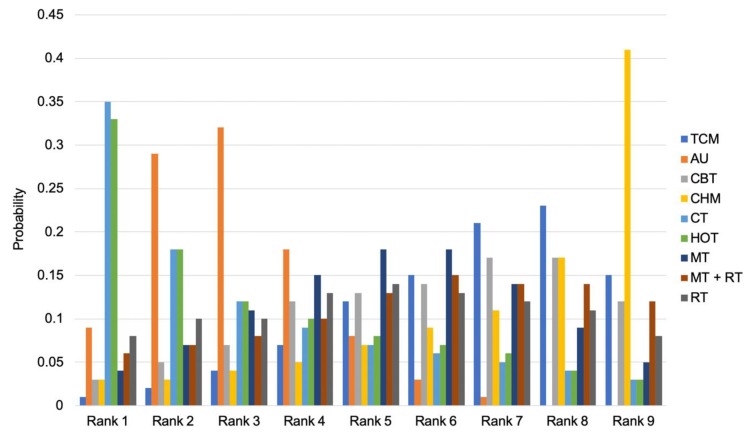
Rank probability of interventions. TCM: traditional Chinese medicine; AU: as usual (treatment, nursing, rehabilitation, education); CBT: cognitive behavioral therapy; CHM: community health management; CT: circuit training; HOT: hyperbaric oxygen therapy; MT: music therapy; RT: respiratory training.

Figure 4 shows the ranking, indicating the probability of being the best intervention to reduce fatigue after stroke, followed by the second best, third best, and so on, among all interventions. As lower fatigue is better, Rank 1 is the worst and the higher cumulative probabilities in Rank 9 indicates better intervention effectiveness. Thus, Rank 9 (in which the cumulative probabilities indicated the best non-pharmacological intervention) was CHM (0.41), rank 8 was TCM (0.23), and rank 7 was CBT (0.17). The worst intervention was rank 1, CT (0.35).

## 4. Discussion

Despite the fact that most interventions did not significantly differ in effectiveness from one another in this review, the cumulative probabilities indicate that the best non-pharmacological intervention for fatigue reduction was CHM, followed by TCM and CBT. The Canadian Stroke Best Practice Recommendations updated the best practice recommendations for PSF in 2019 [35]. Although there is insufficient evidence to recommend pharmacological or non-pharmacological interventions, stroke survivors who experience PSF should be screened and assessed. First, stroke survivors should be routinely asked about PSF during healthcare visits, following return to the community and at transition points. Second, prior to discharge from a hospital, stroke unit, or emergency department, stroke survivors, their families, and informal caregivers should be provided with basic information regarding the frequency and experience of PSF. Third, stroke survivors who experience PSF should be screened for common and treatable post-stroke comorbidities, as well as medications that are associated with and/or exacerbate fatigue. The results also highlight the importance of CHM.

In this review, many interventions could not be included, as there was no control group. One such intervention was COGRAT, an RCT that compares group cognitive therapy (CO) with a new treatment combining cognitive therapy (CO) with graded activity training (GRAT), called COGRAT [36]. Both treatment groups demonstrated significant improvements in fatigue, but a greater proportion of COGRAT participants achieved clinical improvement. As the COGRAT trial had no AU control group, we could not perform network meta-analysis as it was unclear whether the reduction in fatigue was a result of the physical training or a combined effect with CBT. However, we included studies using CBT without supervised exercise therapy, which showed that CBT may be sufficient for clinically significant and sustained improvements in fatigue for at least two months post-treatment [29]. Another study which was excluded as there was no AU control group investigated group therapy versus individual task training [37], where no significant differences between groups were found for improvement of fatigue. We also excluded one study which compared fatigue management (FM) with group stroke education (GSE) [38]. FM was comprised of six psychoeducation sessions aimed at alleviating fatigue, which included an overview and introduction to fatigue, fatigue management, sleep/relaxation, exercise and nutrition, mood, and future focus. Although they reported that FM greatly reduced FSS scores, compared with the GSE group, we could not perform network meta-analysis due to the absence of control intervention. We also found another study that used GSE intervention but could not include it, as the design was a quasi-experiment, not RCT [39]. A previous Cochrane review providing a comprehensive review of PSF intervention [40] showed results that were somewhat similar to ours. It included two non-pharmacological interventions, a fatigue education program, and a mindfulness-based stress reduction program; the results indicated that there was no statistically significant benefit of non-pharmacological intervention and that there was insufficient evidence to show the efficacy of any intervention to treat or prevent PSF. Despite the fact systematic reviews and meta-analyses of randomized trials have long been important synthesis tools for guiding evidence-based medicine, to our knowledge, this is the first network meta-analysis enabling the comparison of multiple non-pharmacological interventions for PSF to incorporate clinical evidence. It could provide evidence for healthcare providers to select effective interventions to improve the health management and quality of life of stroke patients.

This review had several limitations: First, article selection was limited to studies in the English and Chinese languages, which may have introduced a language bias and Ethnic heterogeneity; moreover, the studies were conducted in Australia, the Netherlands, and China, and differences in the prevalence and intervention effectiveness of PSF may be reflected in different countries. However, this study showed good consistency, and more studies are needed to identify the differences among different countries. Second, the sample size and limited data regarding follow-up measurements among the included articles led to an increased heterogeneity between trials. Only two studies had follow-up data, which made a great difference in the result of the endpoint follow-up. However, most previous studies have shown no significant difference in fatigue scores at all time points [41,42]. Third, methodologically, we assessed the risk of bias based on the Cochrane tool, and most trials in this review were judged to be at an unclear or high risk of bias. Thus, we recommend that the results of this study be interpreted with caution. Fourth, we failed to evaluate some important clinical outcomes and comorbidities in PSF patients. In further studies, comorbidities should be considered and assessed. Furthermore, in order to minimize the bias induced by the measurement, we only included FSS and may have missed other interventions. Therefore, future large-sample-sized RCTs based on detailed clinical outcomes may optimize the network and multiple-treatment comparison.

## 5. Conclusions

In conclusion, this network meta-analysis showed no significant differences among fatigue scores in eight PSF non-pharmacological interventions. The cumulative probabilities of best non-pharmacological intervention highlighted CHM followed by TCM and CBT. Despite the high prevalence of fatigue and its great impact on the quality of life in stroke patients, the development of treatment remains compromised due to a lack of understanding by health professionals. Thus, there is an urgent need to recognize PSF, and more accurate assessment methods for PSF need to be developed in order to improve our understanding of its etiology and to develop more effective clinical interventions.

## Figures and Tables

**Table 1:** General fatigue scales.

Scale	Developed By	Target Population	Items
Profile of Mood States—fatigue subscale (POMS)	McNair et al., 1971 [10]	Psychiatric patients	65
Fatigue Severity Scale (FSS)	Krupp et al., 1989 [11]	MS, SLE	9
Fatigue Impact Scale (FIS)	Fisk et al., 1994 [12]	MS, CFS	40
Checklist of Individual Strength (CIS)	Vercoulen et al., 1994 [13]	CFS	24
SF-36 (Vitality subscale)	Ware et al., 1994 [14]	Chronic disease patients	4
Multidimensional Fatigue Inventory (MFI-20)	Smets et al., 1995 [15]	Cancer, CFS, General clinical populations	20
FACIT (Fatigue Scale)	David Cella, et al., 1997 [16]	Chronic Illness	13
Multidimensional Fatigue Symptom Inventory (MFIS)	Stein et al., 1998 [17]	Cancer-related fatigue	6
Brief Fatigue Inventory (BFI)	Tito R et al., 1999 [18]	Cancer-related fatigue	4
Fatigue Assessment Scale (FAS)	Michielsen et al., 2003 [19]	Workers	10
Neurological fatigue index-MS (NFI-MS) in stroke	Mills et al., 2012 [20]	MS	23
Detection List Fatigue (DLF)	Nena Kruithof et al., 2016 [21]	Post-stroke fatigue	9

MS: Multiple sclerosis; SLE: Systemic lupus erythematous; CFS: Chronic fatigue syndrome.

**Table 2:** Network meta-analysis for interventions.

**TCM**								
−1.40 (−3.15, 0.35)	**AU**							
−0.27 (−3.05, 2.61)	1.13 (−1.05, 3.42)	**CBT**						
0.46 (−3.02, 3.87)	1.86 (−1.08, 4.87)	0.71 (−3.09, 4.44)	**CHM**					
−1.61 (−5.19, 1.83)	−0.21 (−3.23, 2.81)	−1.34 (−5.20, 2.24)	−2.07 (−6.35, 2.08)	**CT**				
−1.54 (−5.03, 1.83)	−0.16 (−3.20, 2.85)	−1.29 (−5.09, 2.42)	−1.99 (−6.32, 2.15)	0.05 (−4.24, 4.32)	**HOT**			
−0.59 (−3.28, 2.16)	0.80 (−1.30, 2.95)	−0.32 (−3.46, 2.73)	−1.05 (−4.64, 2.68)	1.01 (−2.62, 4.69)	0.96 (−2.65, 4.80)	**MT**		
−0.39 (−3.64, 2.96)	1.02 (−1.83, 3.94)	−0.08 (−3.94, 3.45)	−0.82 (−5.06, 3.27)	1.23 (−2.95, 5.38)	1.17 (−2.92, 5.37)	0.20 (−2.66, 3.03)	**MT + RT**	
−0.61 (−3.87, 2.71)	0.78 (−2.00, 3.63)	−0.35 (−3.98, 3.25)	−1.08 (−5.16, 3.15)	0.99 (−3.09, 5.13)	0.92 (−3.17, 5.10)	−0.03 (−2.85, 2.80)	−0.24 (−3.29, 2.79)	**RT**

The direct and indirect evidence were mixed comparisons. When the entire 95% confidence interval does not contain 1, the MD is statistically significant. TCM: traditional Chinese medicine; AU: as usual (treatment, nursing, rehabilitation, education); CBT: cognitive behavioral therapy; CHM: community health management; CT: circuit training; HOT: hyperbaric oxygen therapy; MT: music therapy; RT: respiratory training.

## Appendix A

**Table A1:** MEDLINE search terms.

S1	TI stroke or cerebrovascular accident or cva or cerebral vascular event or cve or transient ischemic attack or tia	89,994
S2	TI fatigue or exhaustion or tiredness or lethargy	24,958
S3	TI fatigue after stroke OR TI post stroke fatigue	99
S4	S1 AND S2	195
S5	AB treatment or intervention or therapy or management or rehabilitation	7,697,803
S6	TI controlled clinical trial or randomized controlled trail or randomized or placebo or randomly or trial or groups	478,666
S7	S3 OR S4	195
S8	S5 AND S6 AND S7	7

**Table A2:** Characteristics of all involved studies.

Author/Year	Country	Type of Stroke	Intervention	N			Mean Age (Years)		Time		*p*-Value	
				Gender (Male)	Intervention	Control	Intervention	Control	Post-Incident Stroke	Endpoint	Baseline vs. Post	Baseline vs. Follow-Up
Ingrid G L van de Port, 2012	Netherlands	Ischemic and Haemorrhagic	CT	162	CT = 125	AU = 117	56	58	N/A	24 weeks	>0.05	>0.05
Sylvia Nguyen, 2017	Australia	Ischemic and Haemorrhagic	CBT	11	CBT = 9	AU = 6	47	51	27 months	16 weeks	<0.05	<0.05
Lv Huila, 2017	China	Ischemic and Haemorrhagic	MT	24	MT = 20	AU = 20	62	62	2.28 months	8 weeks	<0.05	N/A
Yin Hongna, 2016	China	Ischemic and Haemorrhagic	TCM	33	AT = 30	AU = 30	62	62	2.95 months	4 weeks	<0.05	N/A
Wang Rongyun, 2017	China	Ischemic and Haemorrhagic	TCM	45	AT = 38	AU = 39	67	67	2 weeks	2 weeks	<0.05	N/A
Li Lanhua, 2014	China	Ischemic and Haemorrhagic	TCM	51	AT = 46	AU = 45	51	51	27days- 20 months	4 weeks	<0.05	N/A
Liu Vanjin, 2018	China	Ischemic and Haemorrhagic	CBT	N/A	CBT = 30	AU = 30	N/A	N/A	N/A	8 weeks	<0.05	N/A
Li Qianfeng, 2017	China	Ischemic	MT RT MT+RT	47	MT = 20 RT = 20 MT + RT = 20	AU = 20	57	57	N/A	8 weeks	<0.05	N/A
Liu Fengli, 2017	China	Ischemic and Haemorrhagic	CHM	43	CHM = 45	AU = 45	47	49	N/A	12 weeks	<0.05	N/A
Zhang Libo, 2014	China	Ischemic and Haemorrhagic	HOT	N/A	HOT = 31	AU = 31	N/A	N/A	N/A	4 weeks	<0.05	N/A

FSS: Fatigue Severity Scale; CT: circuit training; AU: treatment, nursing, rehabilitation as usual; CBT: cognitive behavioral therapy; MT: music therapy; TCM: Traditional Chinese Medicine; RT: respiratory training; CHM: community health management; HOT: hyperbaric oxygen therapy; N/A: not available; there are two p values in the table, the first is FSS scores of baseline vs post and the second is FSS scores of baseline vs follow-up, and only two studies had follow-up data.

## References

[1] Cumming T.B., Packer M., Kramer S.F., English C. (2016). The prevalence of fatigue after stroke: A systematic review and meta-analysis. Int. J. Stroke Off. J. Int. Stroke Soc..

[2] Ingles J.L., Eskes G.A., Phillips S.J. (1999). Fatigue after stroke. Arch. Phys. Med. Rehabil..

[3] Lerdal A., Bakken L.N., Kouwenhoven S.E., Pedersen G., Kirkevold M., Finset A., Kim H.S. (2009). Poststroke Fatigue—A Review. J. Pain Symptom Manag..

[4] Glader E.L., Stegmayr B., Asplund K. (2002). Poststroke fatigue: A 2-year follow-up study of stroke patients in Sweden. Stroke.

[5] Eskes G.A., Lanctôt K.L., Herrmann N., Lindsay P., Bayley M., Bouvier L., Dawson D., Egi S., Gilchrist E., Green T. (2015). Canadian Stroke Best Practice Recommendations: Mood, Cognition and Fatigue Following Stroke practice guidelines, update 2015. Int. J. Stroke.

[6] Rowat A., Pollock A., St George B., Cowey E., Booth J., Lawrence M., Scottish Stroke Nurses Forum (SSNF) (2016). Top 10 research priorities relating to stroke nursing: A rigorous approach to establish a national nurse-led research agenda. J. Adv. Nurs..

[7] Jason L.A., Evans M., Brown M. (2010). What is fatigue? Pathological and non-pathological fatigue. PMR.

[8] De Groot M.H., Phillips S.J., Eskes G.A. (2003). Fatigue Associated with Stroke and Other Neurologic Conditions: Implications for Stroke Rehabilitation. Arch. Phys. Med. Rehabil..

[9] Lynch J., Mead G., Greig C., Young A., Lewis S., Sharpe M. (2007). Fatigue after stroke: The development and evaluation of a case definition. J. Psychosom. Res..

[10] McNair D.M., Lorr M., Droppleman L.F. (1971). Manual for the Profile of Mood States.

[11] Krupp L.B., Alvarez L.A., Larocca N.G., Scheinberg L.C. (1988). Fatigue in multiple sclerosis. Arch. Neurol..

[12] Fisk J.D., Ritvo P.G., Ross L., Haase D.A., Marrie T.J., Schlech W.F. (1994). Measuring the functional impact of fatigue: Initial validation of the Fatigue Impact Scale. Clin. Infect. Dis..

[13] Vercoulen J.H.M.M., Swanink C.M.A., Fennis J.F.M., Galama J.M.D., van der Meer J.W.M., Bleijenberg G. (1994). Dimensional assessment of chronic fatigue syndrome. J. Psychosom. Res..

[14] Ware J.E., Gandek B., The IQOLA Project Group (1994). The SF-36 health survey: Development and use in mental health research and the IQOLA project. Int. J. Mental Health.

[15] Smets E.M., Garssen B., Bonke B., De Haes J.C. (1995). The multidimensional fatigue inventory (MFI) psychometric qualities of an instrument to assess fatigue. J. Psychosom. Res..

[16] Cella D. (1997). Manual of the Functional Assessment of Chronic Illness Therapy (FACIT) Measurement System.

[17] Stein K.D., Martin S.C., Hann D.M., Jacobsen P.B. (1998). A multidimensional measure of fatigue for use with cancer patients. Cancer Pract..

[18] Mendoza T.R., Wang X.S., Cleeland C.S., Morrissey M., Johnson B.A., Wendt J.K., Huber S.L. (1999). The rapid assessment of fatigue severity in cancer patients: Use of the Brief Fatigue Inventory. Cancer.

[19] Michielsen H.J., De Vries J., Van Heck G.L. (2003). Psychometric qualities of a brief self-rated fatigue measure: The Fatigue Assessment Scale. J. Psychosom. Res..

[20] Badaru U.M., Ogwumike O.O., Adeniyi A.F., Olowe O.O. (2013). Variation in functional independence among stroke survivors having fatigue and depression. Neurol. Res. Int..

[21] Kruithof N., Van Cleef M.H., Rasquin S.M., Bovend’Eerdt T.J. (2016). Screening poststroke fatigue; feasibility and validation of an instrument for the screening of poststroke fatigue throughout the rehabilitation process. J. Stroke Cerebrovasc. Dis..

[22] Hinkle J.L., Becker K.J., Kim J.S., McNair N., Choi-Kwon S., Saban K.L., Mead G.E., American Heart Association Council on Cardiovascular and Stroke Nursing and Stroke Council (2017). Poststroke fatigue: Emerging evidence and approaches to management: A scientific statement for healthcare professionals from the American Heart Association. Stroke.

[23] Catalá-López F., Tobías A., Cameron C., Moher D., Hutton B. (2014). Network meta-analysis for comparing treatment effects of multiple interventions: An introduction. Rheumatol. Int..

[24] Hutton B., Salanti G., Caldwell D.M., Chaimani A., Schmid C.H., Cameron C., Ioannidis J.P., Straus S., Thorlund K., Jansen J.P. (2015). The PRISMA extension statement for reporting of systematic reviews incorporating network meta-analyses of health care interventions: Checklist and explanations. Ann. Intern. Med..

[25] Liu F., Li Y., Jiao L., Wang P., Zhao C., Zhou Y., Cheng R. (2017). Effect of community health management on post-stroke fatigue. Chin. J. Prev. Contr. Chron. Dis..

[26] Yin H., Guo Y., Li Q. (2016). Clinical Observation of Acupuncture at Baihui (GV20) and Sishencong (Ex-hn1) Combined with Rehabilitation Training on Patients of Post Stroke Fatigue. JETCM.

[27] Li L., Zheng J. (2014). Observation on integrated traditional Chinese and Western medicine in post-stroke fatigue rehabilitation. World Chin. Med..

[28] Wang R., Lin X., Sun Q. (2017). Clinical Study on Transcutaneous Acupoint Electrical Nerve Stimulation for Post-stroke Fatigue. Shanghai J. Acupunct. Moxibustion.

[29] Nguyen S., Wong D., McKay A., Rajaratnam S.M.W., Spitz G., Williams G., Mansfield D., Ponsford J.L. (2019). Cognitive behavioural therapy for post-stroke fatigue and sleep disturbance: A pilot randomised controlled trial with blind assessment. Neuropsychol. Rehabil..

[30] Liu Y., Wang L., Dong X. (2018). Influence of cognitive behavioral intervention on health behavior and treatment compliance of patients with post stroke fatigue based on problem solving method. Chin. Nurs. Res..

[31] Lv H., Liang W., Liu S., Wang Y., Wang Y. (2017). Effect of music therapy combined with nursing intervention on post-stroke fatigue. Nurs. Pract. Res..

[32] Li Q., Wang Y., Yang W. (2017). Effect of respiratory training instrument combined with music therapy on rehabilitation of post-stroke fatigue. Chin. J. Integr. Med. Cardio-Cerebrovasc. Dis..

[33] van de Port I.G., Wevers L.E., Lindeman E., Kwakkel G. (2012). Effects of circuit training as alternative to usual physiotherapy after stroke: Randomised controlled trial. BMJ.

[34] Libo Z., Xiutang M., Yang W., Xi H., Jie L., Tao L., Xinnian D. (2014). The influence of hyperbaric oxygen therapy on post-stroke fatigue. Chin. J. Conval. Med..

[35] Lanctôt K.L., Lindsay M.P., Smith E.E., Sahlas D.J., Foley N., Gubitz G., Austin M., Ball K., Bhogal S., Blake T. (2019). Canadian Stroke Best Practice Recommendations: Mood, Cognition and Fatigue following Stroke, 6th edition update 2019. Int. J. Stroke.

[36] Zedlitz A.M., Rietveld T.C., Geurts A.C., Fasotti L. (2012). Cognitive and graded activity training can alleviate persistent fatigue after stroke: A randomized, controlled trial. Stroke.

[37] Renner C., Outermans J., Ludwig R., Brendel C., Kwakkel G., Hummelsheim H. (2016). Group therapy task training versus individual task training during inpatient stroke rehabilitation: A randomised controlled trial. Clin. Rehabil..

[38] Clarke A., Barker-Collo S.L., Feigin V.L. (2012). Poststroke Fatigue: Does Group Education Make a Difference? A Randomized Pilot Trial. Top. Stroke Rehabil..

[39] Emery C.E. (2015). Relieving Post-Stroke Fatigue Using a Group-Based Educational Training Approach. Ph.D. Thesis.

[40] Wu S., Kutlubaev M.A., Chun H.Y., Cowey E., Pollock A., Macleod M.R., Dennis M., Keane E., Sharpe M., Mead G.E. (2015). Interventions for post-stroke fatigue. Cochrane Database Syst. Rev..

[41] Christensen D., Johnsen S.P., Watt T., Harder I., Kirkevold M., Andersen G. (2008). Dimensions of Post-Stroke Fatigue: A Two-Year Follow-Up Study. Cerebrovasc. Dis..

[42] Snaphaan L., van der Werf S., de Leeuw F.E. (2011). Time course and risk factors of post-stroke fatigue: A prospective cohort study. Eur. J. Neurol..

